# Good Remedies—Out of Fashion

**Published:** 1883-12

**Authors:** 


### Notes on Books.

---

*Good Remedies—out of Fashion.* By CHARLES J. HARE, M.D. Cantab., F.R.C.P. London: J. & A. Churchill. 1883.

Though elevated to the dignity of book-form this reprint of Dr. Hare's address to the Metropolitan Counties Branch of the British Medical Association would, in respect of quantity, more fittingly have been issued as a *brochure*. But, voluminous though it be not, it yet affords food for reflection upon the mutability of fashion in medical treatment. Dr. Hare's experience and knowledge are extensive, and he ought to be intimately acquainted with the relative prevalence of various methods of treatment; but we should not have thought that the use of emetics in Croup, Bronchitis, and disordered gastric secretion was so completely extinct as he considers it to be. So also of Opium he states—we hope truly—"it was formerly much more used than now." If this be indeed the case, it can only be because Opium and its derivatives have been more or less replaced by other narcotics, not because the ever increasing struggle for daily existence renders less frequent the necessity for employing nervine sedatives.

Something like a moiety of the lecture is devoted to the consideration of "Bleeding in one form or another," which, in Dr. Hare's opinion, "has more than any other means whatever the effect of assuaging pain, of modifying the course of diseases and of saving life." This Dr. Hare "steadfastly believes," in part because "the united testimony of the sages of our profession for more than 2,000 years bore witness to" its trustworthiness. For our own part we cannot agree that any article of belief should be accepted as true on such grounds. Do we still think the arteries are passages for air? The ancient doctrine of Signatures was still so firmly held in More's day that he based upon it his proof of the existence of an intelligent Creator, and to discredit it he considered was to be an atheist. Where is that opinion now? The possession of aphrodisiac and semi-human properties by the mandrake was universally believed from before the time of Rachel until after the age of Shakspere, more than 3,000 years. Sir T. Browne thought those properties, if they existed, little short of miraculous; are they now thought anything but fabulous? On the other hand, however, it is only fair to remember that confidence in the reputed benefits of bleeding still continued to be had after medicine had begun to be placed upon a scientific basis of investigation and experiment, and the practice must not therefore be discarded without receiving at the hands of intelligent practitioners an unbiassed employment in the treatment of certain diseased conditions. One cannot but be forcibly struck by the rapid and complete revulsion from the free use of leeches which took place between the years 1842 and 1862, as brought out by Dr. Hare's statistics. If, with our author, we agree to abandon the theory of change of type of disease to explain the change of plan of treatment, we have nothing else to fall back upon for an explanation but the recoil from that opinion which, with some exaggeration, Le Sage puts into the mouth of Sangrado:—"Sçaches, mon ami, qu'il ne faut que saigner, et faire boire de l'eau chaude. Voilà le secret de guérir toutes les maladies du monde. Ce simple secret que je te révèle est renfermé dans ces deux points, dans la saignée et dans la boisson fréquente. Je n'ai plus rien à t'apprendre: tu sçais la médecine à fonds."

Our profession has never perhaps been more severely ridiculed than by Molière, but the perusal of this address vividly recalls Sganarelle's words:—"Oui cela était autrefois ainsi; mais nous avons changé tout cela, et nous faisons maintenant la médecine d'une méthode toute nouvelle." Perhaps in our capricious and utter abandonment of venesection we are no more right than "le médecin malgré lui" was in his ideas as to the anatomical relations of the Heart and Liver. Perhaps, also, now that port wine is again coming into vogue, we shall again witness the modes of treatment of which it was formerly the social contemporary. We commend Dr. Hare's book to our readers' thoughtful perusal.

